# Supplementary material for: fMRI repetition suppression reveals no sensitivity to trait judgments from faces in face perception or theory-of-mind networks
Source: PLoS One. 2018 Aug 14;13(8):e0201237. doi: 10.1371/journal.pone.0201237 (PMC6091917; doi:10.1371/journal.pone.0201237)
Supplement: S1 Method — (DOCX) [file pone.0201237.s001.docx]

**Supplementary Method**

**Pilot experiment to evaluate stimuli**

**Participants**

Fourteen participants completed the pilot experiment (8 female, mean age = 25.14, SD = 9.52). All participants received course credit, had normal or corrected-to-normal vision and gave informed consent according to the local ethics guidelines.

**Design**

We piloted the stimuli using two self-paced tasks, a two-alternative forced-choice task (2AFC) and a ratings task. Participants completed the 2AFC task before the ratings task. Across participants, 16 relevant statements from the mini-IPIP (4 per trait) and all 434 face images were used.

**Tasks**

**2AFC***.* On each trial, a Low and High trait image on the same ID were presented side by side. Trials were presented in blocks with a trait statement and task instructions presented at the beginning of each block (Supplementary Figure 1). Blocks included 26, 27 or 28 trials depending on the number of stimuli available per trait category and blocks were randomly ordered. Each ID pair was presented twice throughout the experiment, so that two trait relevant statements could be presented per ID per participant. Half of the participants were shown statements from list 1, the other half were shown statements from list 2 (Supplementary Table 2). On each trial, the choice of which stimulus matched the statement was recorded. All 217 ID pairs were presented, each with 2 statements, which made 434 trials in total. For each trait type, a one-tailed one-sample t-test was performed to test if judgments were greater than chance performance (50%) and thereby confirm if stimuli were perceived in a manner that we expected. Cohen’s d_z_ was also calculated as a standardised measure of effect size.

**Ratings***.* On each trial, a single face image was presented in the centre of the screen and participants were asked to make a judgement based on a statement presented at the start of the block. Trials were presented in blocks with a trait statement and task instructions presented at the beginning of each block (Supplementary Figure 1). Participants responded on a keyboard number pad using the numbers one to nine, where one indicated that the statement presented at the beginning of the block suited the face “not at all well” and nine indicated that it fitted “extremely well”. Half of the participants saw the first half of the stimulus IDs warped to a High trait dimension and the second half of the IDs warped to a Low trait dimension. The remaining participants saw the reverse. In addition, the first half of these participant groups were asked to rate faces according to statement list 1, whereas the second half of the participant groups were asked to rate faces according to statement list 2. This resulted in 4 different orders so that one participant sees high with first statement list, next sees low with first statement list, third sees high with second statement list, fourth sees low with second statement list. Thus, across participants, all face IDs were rated on each relevant statement from the mini-IPIP when warped to high or to low of the relevant trait composite. In addition, no participant saw the same ID warped to high in the ratings section if they had seen it warped to low in the ratings section, and vice versa. Each participant was shown each exemplar twice with a different trait statement each time, which produced 434 trials in total. Ratings for high and low faces for each trait were compared using a one-tailed paired samples t-test and Cohen’s d_z_ as a measure of effect size.

**Results**

**2AFC***.* Except for the agreeableness pairs, all other pairs were discriminated correctly at a level above chance (Supplementary Figure 2A; Supplementary Table 3). Each trait is compared to chance performance (50%) with a one-tailed, one-sample t-test and Cohen’s d_z_: Extraversion t(13)= 7.95, p<.001, d_z_ = 2.13; Agreeableness t(13)= -0.86, p=.797, dz = -0.23; Neuroticism t(13)= 5.53, p<.001, d_z_ = 1.48; Physical Health t(13)= 3.92, p<.001, d_z_ = 1.05.

**Ratings***.* Except for the agreeableness stimuli, all other high/low trait pairs were rated significantly different from each other, with the high warp rated higher in each trait than the low warp (Supplementary Figure 2B; Supplementary Table 3). Ratings for high and low faces for each trait were compared using a one-tailed paired samples t-test and Cohen’s dz: Extraversion t(13)= 4.24, p<.001, d_z_ = 1.13; Agreeableness t(13)= -0.98, p=.826, d_z_ = -0.26; Neuroticism t(13)= 5.22, p<.001, d_z_ = 1.39; Physical Health t(13)= 3.04, p=.005, d_z_ = 0.81.
