# Supplementary material for: fMRI repetition suppression reveals no sensitivity to trait judgments from faces in face perception or theory-of-mind networks
Source: PLoS One. 2018 Aug 14;13(8):e0201237. doi: 10.1371/journal.pone.0201237 (PMC6091917; doi:10.1371/journal.pone.0201237)
Supplement: S1 Table — Average self-reported trait scores for individuals included in the high and low composites, as well as individuals before transformation. As should be the case, average scores are different for individuals included in the high and low composites (no overlap of 95%CIs, in square brackets). In addition, average scores for individual ratings that would later be transformed do not overlap with average scores from the high or low composites (no overlap of 95% CIs). The lack of overlap between individual images and composite images suggests that prior to transformation individuals are in a neutral position, not especially skewed towards those included in the high or the low composites images. (DOCX) [file pone.0201237.s002.docx]

**S1 Table.** Average self-reported trait scores

|  | High Composite | Low Composite | Individuals |
| --- | --- | --- | --- |
| Extraversion | 4.69 [4.61, 4.77] | 2.08 [1.90, 2.27] | 3.54 [3.40, 3.68] |
| Agreeableness | 4.70 [4.62, 4.77] | 2.66 [2.51, 2.81] | 3.89 [3.79, 3.99] |
| Neuroticism | 4.51 [4.40, 4.62] | 1.73 [1.55, 1.90] | 3.10 [2.95, 3.25] |
| Physical Health | 59.95 [59.00, 60.89] | 40.61 [38.26, 42.95] | 52.88 [51.95, 53.81] |

Average self-reported trait scores for individuals included in the high and low composites, as well as individuals before transformation. As should be the case, average scores are different for individuals included in the high and low composites (no overlap of 95%CIs, in square brackets). In addition, average scores for individual ratings that would later be transformed do not overlap with average scores from the high or low composites (no overlap of 95% CIs). The lack of overlap between individual images and composite images suggests that prior to transformation individuals are in a neutral position, not especially skewed towards those included in the high or the low composites images.
