# Supplementary material for: fMRI repetition suppression reveals no sensitivity to trait judgments from faces in face perception or theory-of-mind networks
Source: PLoS One. 2018 Aug 14;13(8):e0201237. doi: 10.1371/journal.pone.0201237 (PMC6091917; doi:10.1371/journal.pone.0201237)
Supplement: S2 Table — Note: For items with a number 1 in the reverse score column, subjects’ scores were reversed so that a high score represents high trait representation. (DOCX) [file pone.0201237.s003.docx]

**S2 Table.** Statements used in the pilot experiment.

|  | **Trait Type** | **Reverse Score** |
| --- | --- | --- |
| **Statement List 1** |  |  |
| More sympathetic | Agreeableness | 0 |
| Not interested in other people's problems | Agreeableness | 1 |
| Is the life of the party | Extraversion | 0 |
| Doesn't talk a lot | Extraversion | 1 |
| Has frequent mood swings | Neuroticism | 0 |
| Is relaxed most of the time | Neuroticism | 1 |
| Health is good | Physical Health | 0 |
| Accomplishes less due to health problems | Physical Health | 1 |
|  |  |  |
| **Statement List 2** |  |  |
| Feels others' emotions | Agreeableness | 0 |
| Not really interested in others | Agreeableness | 1 |
| Talks to a lot of different people at parties | Extraversion | 0 |
| Keeps in the background | Extraversion | 1 |
| Gets upset easily | Neuroticism | 0 |
| Seldom feels blue | Neuroticism | 1 |
| Finds it easy to climb the stairs | Physical Health | 0 |
| Pain interferes more with work | Physical Health | 1 |

Note: For items with a number 1 in the reverse score column, subjects’ scores were reversed so that a high score represents high trait representation.
