# Supplementary material for: fMRI repetition suppression reveals no sensitivity to trait judgments from faces in face perception or theory-of-mind networks
Source: PLoS One. 2018 Aug 14;13(8):e0201237. doi: 10.1371/journal.pone.0201237 (PMC6091917; doi:10.1371/journal.pone.0201237)
Supplement: S3 Table — Square brackets = 95% Confidence intervals. (DOCX) [file pone.0201237.s004.docx]

**S3 Table.** Results from the two-alternative forced-choice task (2AFC) and the ratings task.

|  | 2AFC | Rating high | Rating low |
| --- | --- | --- | --- |
| Extraversion | 81.08 [73.42, 88.75] | 5.58 [5.28, 5.88] | 4.68 [4.32, 5.04] |
| Agreeableness | 46.90 [39.82, 53.98] | 5.07 [4.83, 5.30] | 5.19 [5.01, 5.38] |
| Neuroticism | 76.21 [66.92, 85.50] | 5.29 [5.11, 5.48] | 4.51 [4.31, 4.70] |
| Physical Health | 71.76 [60.87, 82.65] | 5.69 [5.39, 6.00] | 5.10 [4.68, 5.52] |
| Overall | 69.16 [65.16, 73.15] | 5.41 [5.26, 5.56] | 4.86 [4.69, 5.04] |

Square brackets = 95% Confidence intervals
