# Supplementary material for: fMRI repetition suppression reveals no sensitivity to trait judgments from faces in face perception or theory-of-mind networks
Source: PLoS One. 2018 Aug 14;13(8):e0201237. doi: 10.1371/journal.pone.0201237 (PMC6091917; doi:10.1371/journal.pone.0201237)
Supplement: S4 Table — Abbreviations: ROI = Region of interest; fdr = false discovery rate; OFA = occipital face area; FFA = right fusiform face area; pSTS = posterior superior temporal sulcus; TPJ = temporoparietal junction; mPFC = medial prefrontal cortex; ant. Temp. = anterior temporal; MTG = middle temporal gyrus; MFG = middle frontal gyrus; OT = occipitotemporal cortex. Note: ‘ROI size’ is the total number of voxels in each ROI based on data from a face perception localiser or a theory-of-mind localiser. ‘Average localiser mask size’ is the number of voxels that overlap in more than 50% of participants within each ROI. Right MTG, for example, consists of a 200 voxel ROI, with 38 voxels showing overlap in 82% of participants. Analyses were performed on the subset of voxels in each ROI that show overlap in a majority of participants (>50%). (DOCX) [file pone.0201237.s005.docx]

**S4 Table.** Exploratory analysis of wider face perception and theory of mind networks.

| **Region** |  |  |  | **Novel>Repeated** | | |
| --- | --- | --- | --- | --- | --- | --- |
|  | **ROI size (voxels)** | **Average localiser mask size (voxels)** | **Inter-subject overlap (%)** | **Percent signal change (SEM)** | **t** | **p(fdr)** |
| *Face localiser* |  |  |  |  |  |  |
| Right MTG | 200 | 38 | 82 | .135 (.01) | .50 | .82 |
| Left OFA | 177 | 32 | 75 | .050 (.21) | .41 | .82 |
| Right OT cortex | 56 | 12 | 54 | -.199 (.25) | -.76 | .82 |
| Left MTG | 141 | 24 | 64 | .013 (.20) | .07 | .82 |
| Left pSTS | 140 | 23 | 75 | -.107 (.19) | -.57 | .82 |
| Left FFA | 57 | 9 | 57 | .380 (.22) | 1.75 | .51 |
|  |  |  |  |  |  |  |
| *ToM localiser* |  |  |  |  |  |  |
| Precuneus | 870 | 206 | 96 | -.086 (.14) | -.62 | .80 |
| Left TPJ | 615 | 143 | 100 | .129 (.14) | .92 | .80 |
| Left ant. temp. cortex | 139 | 27 | 75 | .151 (.11) | 1.35 | .80 |
| Right MFG | 74 | 13 | 57 | -.049 (.13) | -.37 | .80 |
| Left MFG | 31 | 4 | 54 | -.049 (.25) | -.20 | .80 |

Abbreviations: ROI = Region of interest; fdr = false discovery rate; OFA = occipital face area; FFA = right fusiform face area; pSTS = posterior superior temporal sulcus; TPJ = temporoparietal junction; mPFC = medial prefrontal cortex; ant. Temp. = anterior temporal; MTG = middle temporal gyrus; MFG = middle frontal gyrus; OT = occipitotemporal cortex.

Note: ‘ROI size’ is the total number of voxels in each ROI based on data from a face perception localiser or a theory-of-mind localiser. ‘Average localiser mask size’ is the number of voxels that overlap in more than 50% of participants within each ROI. Right MTG, for example, consists of a 200 voxel ROI, with 38 voxels showing overlap in 82% of participants. Analyses were performed on the subset of voxels in each ROI that show overlap in a majority of participants (>50%).
