# Supplementary figures and images for: fMRI repetition suppression reveals no sensitivity to trait judgments from faces in face perception or theory-of-mind networks
Source: PLoS One. 2018 Aug 14;13(8):e0201237. doi: 10.1371/journal.pone.0201237 (PMC6091917; doi:10.1371/journal.pone.0201237)

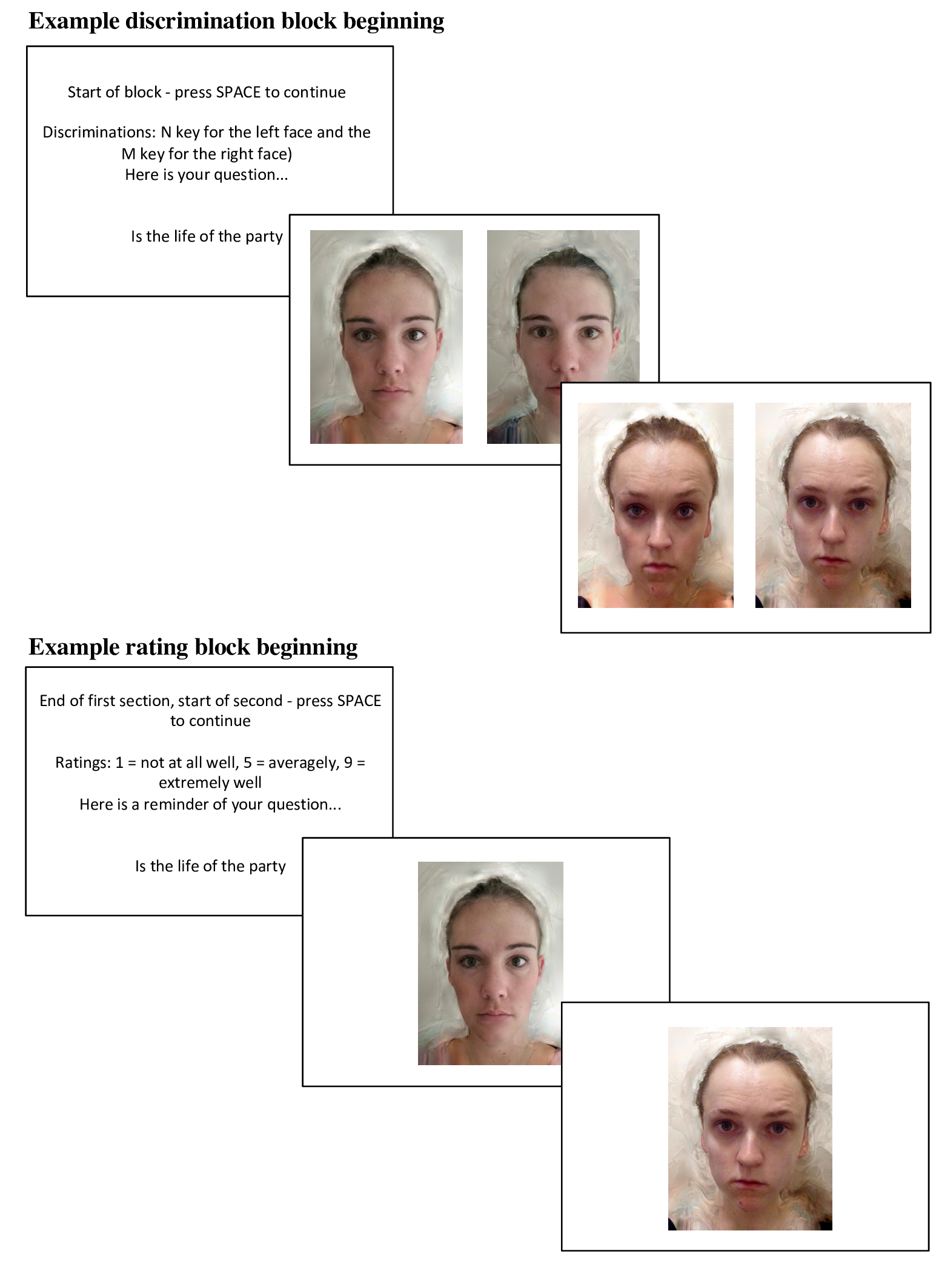

Supplement: S1 Fig — Note: The images used are for illustrative purposes and were not used in the pilot experiment. (TIFF) [file pone.0201237.s006.tiff]

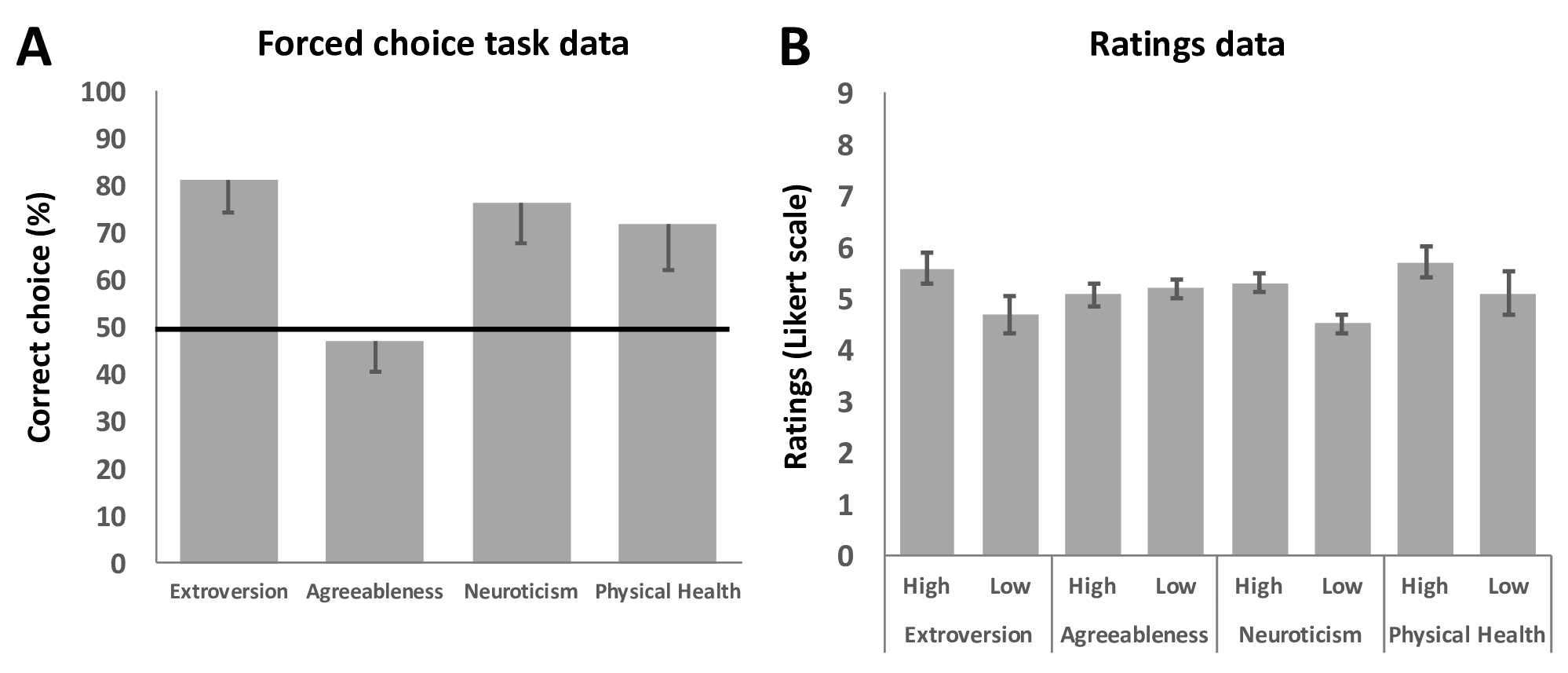

Supplement: S2 Fig — Face judgment data in a two-alternative forced-choice task (A) and a ratings task (B). The black line at 50% in (A) represents chance performance. Error bars are 95% confidence intervals. One-tailed confidence intervals are displayed in (A) to reflect the one-tailed hypothesis in each comparison. (TIFF) [file pone.0201237.s007.tiff]
